# Microwave-Assisted Hydrothermal Rapid Synthesis of Calcium Phosphates: Structural Control and Application in Protein Adsorption

**DOI:** 10.3390/nano5031284

**Published:** 2015-07-31

**Authors:** Zhu-Yun Cai, Fan Peng, Yun-Peng Zi, Feng Chen, Qi-Rong Qian

**Affiliations:** 1 Department of Orthopedics, Changzheng Hospital, Second Military Medical University, Shanghai 200003, China; E-Mails: caizhuyun@126.com (Z.-Y.C.); maspf@163.com (F.P.); ziyp0935@163.com (Y.-P.Z.); 2 State Key Laboratory of High Performance Ceramics and Superfine Microstructure, Shanghai Institute of Ceramics, Chinese Academy of Sciences, Shanghai 200050, China

**Keywords:** hydroxyapatite, microwave, nanostructure, drug delivery, protein adsorption

## Abstract

Synthetic calcium phosphate (CaP)-based materials have attracted much attention in the biomedical field. In this study, we have investigated the effect of pH values on CaP nanostructures prepared using a microwave-assisted hydrothermal method. The hierarchical nanosheet-assembled hydroxyapatite (HAP) nanostructure was prepared under weak acidic conditions (pH 5), while the HAP nanorod was prepared under neutral (pH 7) and weak alkali (pH 9) condition. However, when the pH value increases to 11, a mixed product of HAP nanorod and tri-calcium phosphate nanoparticle was obtained. The results indicated that the pH value of the initial reaction solution played an important role in the phase and structure of the CaP. Furthermore, the protein adsorption and release performance of the as-prepared CaP nanostructures were investigated by using hemoglobin (Hb) as a model protein. The sample that was prepared at pH = 11 and consisted of mixed morphologies of nanorods and nanoprisms showed a higher Hb protein adsorption capacity than the sample prepared at pH 5, which could be explained by its smaller size and dispersed structure. The results revealed the relatively high protein adsorption capacity of the as-prepared CaP nanostructures, which show promise for applications in various biomedical fields such as drug delivery and protein adsorption.

## 1. Introduction

Calcium phosphate (CaP) materials have attracted much attention in the biomedical field, due to their excellent biocompatibility [1,2,3]. In native mineral tissue, nanostructured CaP can self-assemble into hierarchical structures to achieve excellent biological functions [4,5,6]. The synthetic CaP nanomaterials which have the similar chemical structure to the native mineral component in the bones and teeth of vertebrates are usually considered as an ideal biomaterial [7,8]. The synthetic CaP nanomaterials are deemed to be good for biocompatibility and bioactivity, and have been widely investigated in various applications, including drug delivery [9], protein adsorption [10], bone defect repair/tissue engineering [11,12], and other biomedical areas [13,14].

CaP-based materials include many kinds of chemical phases with different structure and properties, including octacalcium phosphate (Ca_8_(HPO_4_)_2_(PO_4_)_4_·5H_2_O), α-tricalcium phosphate (α-Ca_3_(PO_4_)_2_), β-tricalcium phosphate (β-Ca_3_(PO_4_)_2_), amorphous calcium phosphate (Ca*_x_*H*_y_*(PO_4_)*_z_*·*n*H_2_O, *n* = 3–4.5, 15%–20% H_2_O), hydroxyapatite (Ca_10_(PO_4_)_6_(OH)_2_), fluorapatite (Ca_10_(PO_4_)_6_F_2_), and so on [15,16]. The crystal phase and morphology of synthetic CaP are strongly influenced by the preparation methods. In recent decades, a variety of CaP based materials with different morphologies, including nanorods, plate-like nanocrystals, nanoparticles, nanotubes and three-dimensional structures, have been prepared using different methods [8]. For example, various HAP materials have been prepared by solvothermal method, such as hierarchical microspheres [17], ordered arrays [18], nanoparticles [19], *etc.*

Compared to conventional heating methods, microwave heating technology is emerging as a form of rapid volumetric heating with advantages such as speed, shorter reaction time (usually in minutes), high efficiency, and energy savings [20]. Microwave heating has no thermal gradients throughout the bulk media and leads to efficient and uniform reactions [20,21]. Therefore, microwave heating technology has become a fast-growing area of research in synthetic chemistry, and has been widely used in the synthesis of various inorganic nanostructured materials including CaP biomaterials [22,23]. Up to now, various CaP nanostructured materials have been prepared by the microwave-assisted method in liquid phase, including nanoparticles and one-, two- and three-dimensional nanostructures [1,24,25,26].

The preparation, control over structure/size/morphology, properties and applications of CaP nanostructures have become a hot topic in the biomedical research field. Herein, we investigate the influence of pH value on the microwave-assisted hydrothermal rapid synthesis of CaP nanostructures. The HAP nanosheet-assembled hierarchical nanostructure, HAP nanorod and tri-calcium phosphate (TCP) nanoparticles were prepared with different pH values for the initial reaction solutions. Then, the as-prepared CaP nanostructures are explored for application in protein adsorption/release with hemoglobin (Hb) as a model protein, which exhibits a relatively high protein adsorption capacity and sustained release properties. The as-prepared CaP nanostructures are promising for applications in various biomedical fields such as drug delivery and protein adsorption.

## 2. Results and Discussion

**Figure 1:**
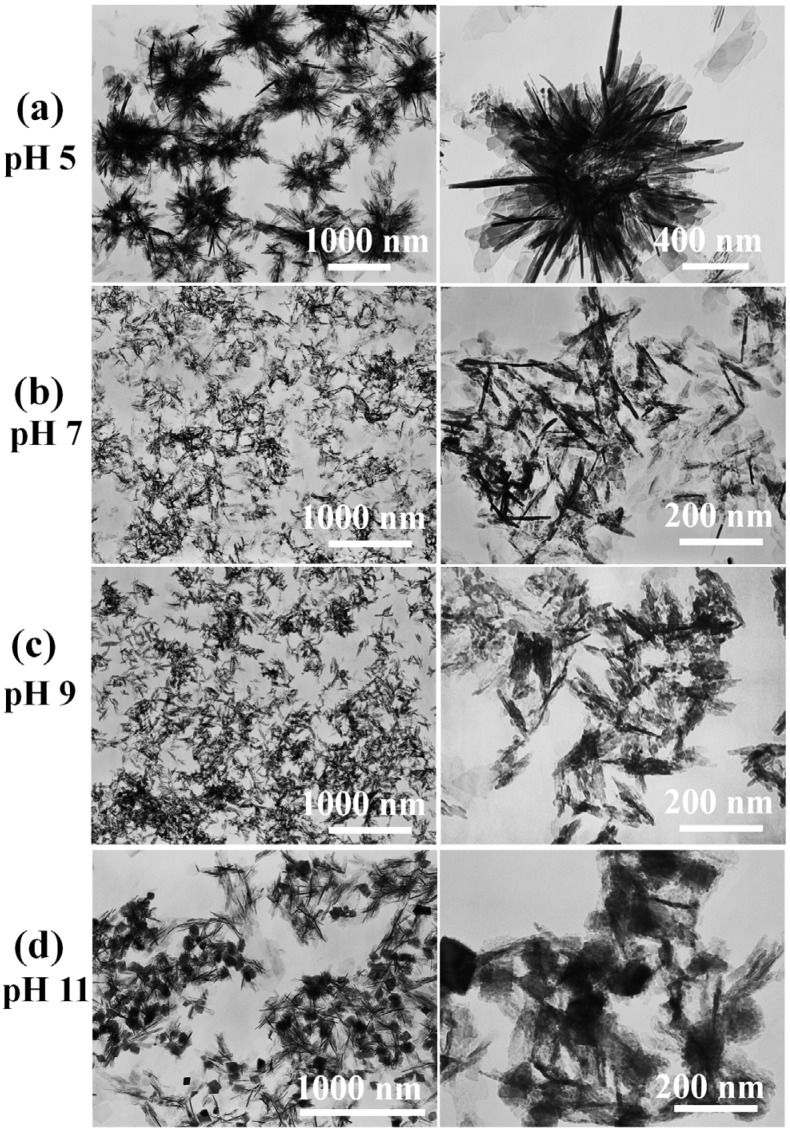
SEM of calcium phosphate nanostructures prepared with the microwave-assisted hydrothermal method under different pH values: (**a**) Sample A, pH = 5; (**b**) Sample B, pH = 7; (**c**) Sample C, pH = 9; (**d**) Sample D, pH = 11.

As shown in Figure 1a, the product, which was prepared using a microwave-assisted hydrothermal method at pH = 5, consisted of HAP nanosheet-assembly flower-like hierarchical nanostructures, and the thickness of the HAP nanosheet was about 10 nm. When the pH values of the initial reaction solution increase to 7 and 9, the products prepared under the same conditons consisted of HAP nanorods with a length of about 100 nm (Figure 1b,c). However, the product consisted of mixed morphologies of nanorods and nanoprisms (Figure 1d) when the pH value of the initial reaction solution was further increased to 11. These results indicate that the pH value has an important influence on the morphology and structure of CaP materials prepared with the microwave-assisted hydrothermal method.

**Figure 2:**
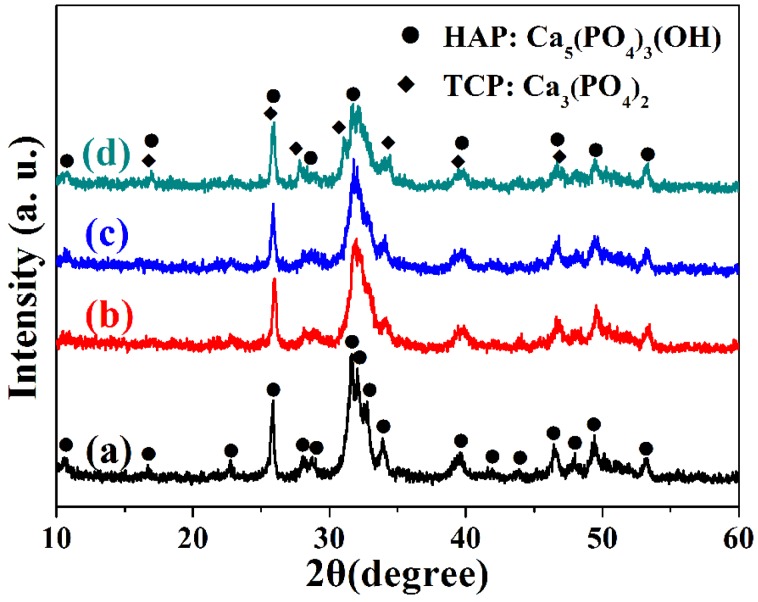
XRD patterns of calcium phosphate nanostructures prepared by microwave-assisted hydrothermal method under different pH values: (**a**) Sample A, pH = 5; (**b**) Sample B, pH = 7; (**c**) Sample C, pH = 9; (**d**) Sample D, pH = 11.

The crystal phases of the CaP nanostructures prepared with the microwave-assisted hydrothermal method have been characterized by X-ray powder diffraction (XRD). As shown in Figure 2a–c, the products of samples A, B and C consisted of HAP (JCPDS No. 09-0432) when the pH values of the initial solution were adjusted to 5, 7 and 9. However, the as-prepared sample consisted of a mixture of HAP and tri-calcium phosphate (TCP, JCPDS No. 09-0169) (Figure 2d), when the pH value increase to 11. These results indicate that the pH value of the reaction solution has an obvious effect on the crystal phase of CaP nanostructures prepared by microwave-assisted hydrothermal method.

**Figure 3:**
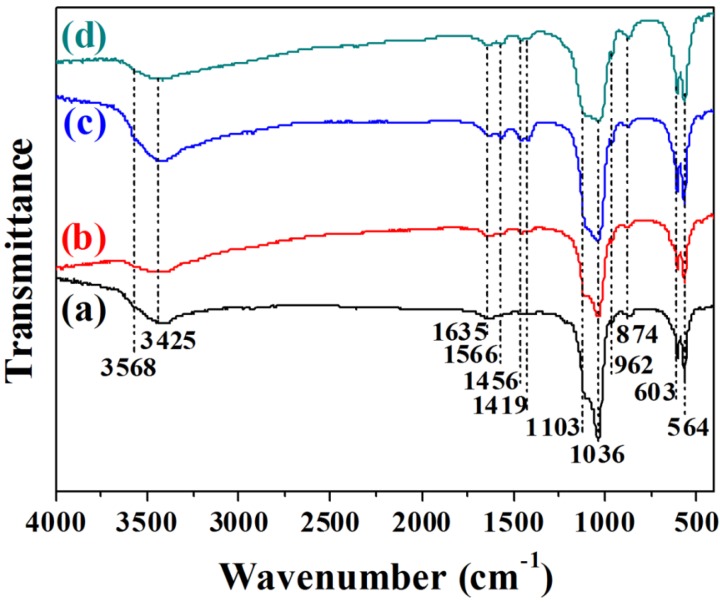
FTIR spectra of calcium phosphate nanostructures prepared by microwave-assisted hydrothermal method under different pH values: (**a**) Sample A, pH = 5; (**b**) Sample B, pH = 7; (**c**) Sample C, pH = 9; (**d**) Sample D, pH = 11.

To further investigate the chemical properties of the samples, the Fourier transform infrared (FTIR) spectra of the as-prepared samples by microwave-assisted hydrothermal method under different pH values were measured. As shown in Figure 3, the FTIR spectra exhibit similar absorption bands. The broad absorption bands at around 3425 and 1635 cm^−1^ are assigned to the adsorbed water. The absorption band at about 3568 cm^−1^ arises from the hydroxyl group in HAP. The absorption bands at 1103, 1034, 962, 603 and 564 cm^−1^ are attributed to PO_4_^3−^ ions in samples. Among these peaks, the absorptions at 1103 and 1034 cm^−1^ are assigned to the ν_3_ vibrations of the P–O bond of the phosphate group [27]. In addition, the samples exhibit the ν_3_ vibrational mode of carbonate ion at 1456 and 1419 cm^−1^, as well as the ν_2_ vibrational mode of carbonate ion at 874 cm^−1^, which is assigned to the (CO_3_)^2−^ group of B-type HAP [28,29]. The absorption band at 1566 cm^−1^ is associated with the (CO_3_)^2−^ substituted for OH^−^ as A-type substitution [30]. The presence of (CO_3_)^2−^ group in the HAP lattice may be derived from the dissolved CO_2_ from atmosphere.

**Figure 4:**
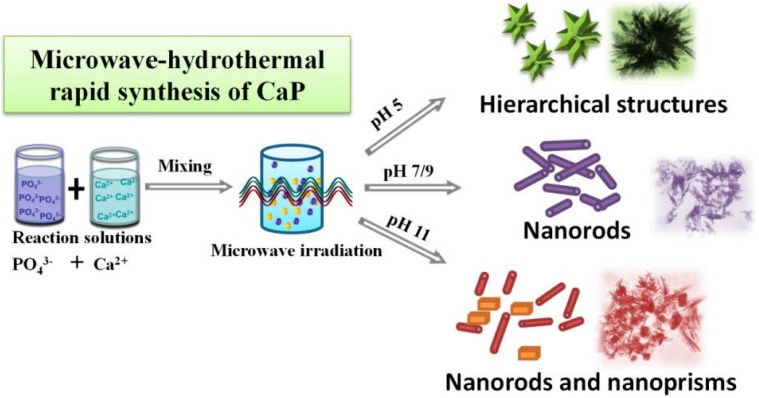
Schematic illustration for the synthesis of calcium phosphate nanostructures with the microwave-assisted hydrothermal method under different pH values.

The schematic illustration for the preparation of CaP nanostructures is shown in Figure 4. We investigate the influence of pH value on the CaP nanostructures synthesized by a microwave-assisted rapid hydrothermal method at 120 °C for 10 min. The HAP nanosheet-assembled hierarchical nanostructures can be prepared under a weak acidic conditions (pH 5), while the HAP nanorods can be prepared under a neutral (pH 7) and weak alkali conditions (pH 9). When the pH value increase to 11, the product consisted of a mixture of HAP nanorods and tri-calcium phosphate (TCP) nanoparticles. These results indicate that the pH value plays an important role in controlling the structure and chemical properties of CaP prepared with the microwave-assisted hydrothermal method.

A possible mechanism is proposed to illustrate the formation process of the HAP nanosheet-assembly flower-like hierarchical nanostructure. Due to the low pH value in the initial reaction solution, the amorphous CaP nuclei may appear due to the reaction in the precursor solution. Then, the temperature increased rapidly in a microwave-assisted hydrothermal environment. The amorphous CaP nuclei cannot guide the direction of crystallization and crystal growth at the initial stage [31]. Therefore, the products of spherulites composed of HAP nanosheets organized in a radial orientation are obtained, due to the disordered crystallization of amorphous CaP nuclei and crystal growth using the abundant Ca^2+^ and PO_4_^3−^ ions in the reaction solution, under microwave-assisted hydrothermal conditions.

On the other hand, the HAP nanorods are prepared when the pH values of the reaction solution increase to 7 and 9. This result can be explained by the different reaction mechanism compared with the HAP nanosheet-assembly hierarchical nanostructure prepared with the microwave-assisted hydrothermal method at a lower pH value (pH = 5). In the precursor solution with high pH values (7 and 9), a large number of CaP nuclei with low crystallinity may appear due to the reaction of abundant Ca^2+^ and PO_4_^3−^ ions in the initial reaction solution. Thereafter, when the reaction solutions are transformed to a microwave-assisted hydrothermal environment, the product of HAP nanorods can occur via a crystal growth process with the templates of these CaP nuclei, and there is a specific preferred growth direction along the *c*-axis of the hexagonal HAP. Therefore, the HAP nanorods can be prepared at the pH values of 7 and 9.

When the pH value is increased to 11, the as-prepared CaP product consisted of a mixture of HAP nanorods and TCP nanoprisms. We propose that the presence of TCP phase in the product may be explained by the combined effect of the high pH value and the hydrothermal environment. In the precursor solution with high pH values (pH = 11), the CaP nuclei with crystal phase of HAP and TCP may appear due to the reaction of abundant Ca^2+^ and PO_4_^3^^−^ ions. Then, the product of HAP nanorods and TCP nanoprisms can be formed through the crystal growth of these CaP nuclei in the microwave-assisted hydrothermal environment. The TCP and HA materials are usually selected and mixed to prepare composite materials which can be used as important biomaterials for bone defect repair and other biomedical fields, due to their high biocompatibility and controllable biodegradability [32,33]. In this study, the composite material of nanostructured TCP and HA can be directly and rapidly prepared with the microwave-assisted hydrothermal method under a lower temperature. This strategy may be favorable for the preparation of homogenized TCP/HA composite materials.

**Figure 5:**
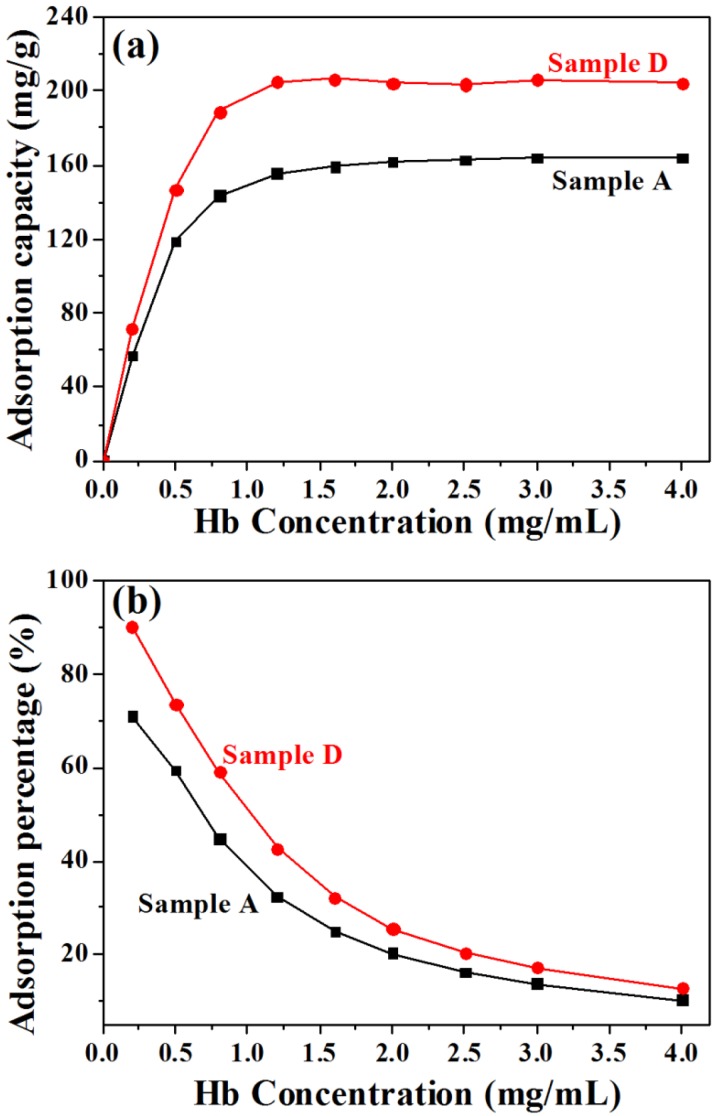
The Hb protein adsorption properties of Sample A (pH 5) and Sample D (pH 11). (**a**) Hb adsorption capacity as a function of initial Hb concentration; (**b**) Hb adsorption percentage as a function of initial Hb concentration.

We have also investigated the protein adsorption performance of Sample A and Sample D by using Hb as a model protein. The Hb adsorption amounts for both Sample A and Sample D increase with increasing initial Hb concentration from 0.2 to 1.2 mg·mL^−1^, and separately reach a plateau of 155 and 205 mg·g^−1^ at a Hb concentration of 1.2 mg·mL^−1^ (Figure 5a). However, the Hb adsorption percentages decrease from 71% to 10% for Sample A, and decrease from 90% to 13% for Sample D, with increasing Hb initial concentration from 0.2 to 4.0 mg·mL^−1^ (Figure 5b).

The high protein adsorption properties of the as-prepared CaP nanostructures can be explained by the native chemical properties of CaP based materials. The native bone tissue constructed by the CaP materials and collagen is a native reservoir for storing growth factors, fatty acids, and other biomolecules [34]. Therefore, we think CaP-based materials have the advantage of high adsorption capacity for a variety of biomolecules, due to a high binding affinity. Up to now, CaP based materials have been used as a delivery vehicle for various proteins, growth factors, antibiotics, macromolecules and so on [35,36,37].

The Langmuir adsorption model is used to calculate the maximum Hb adsorption capacity of the as-prepared CaP nanostructures:

(1) $$q_{e}=\frac{q_{m}bc_{e}}{1+bc_{e}}$$

(2) $$\frac{c_{e}}{q_{e}}=\frac{1}{q_{m}b}+\frac{c_{e}}{q_{m}}$$

**Figure 6:**
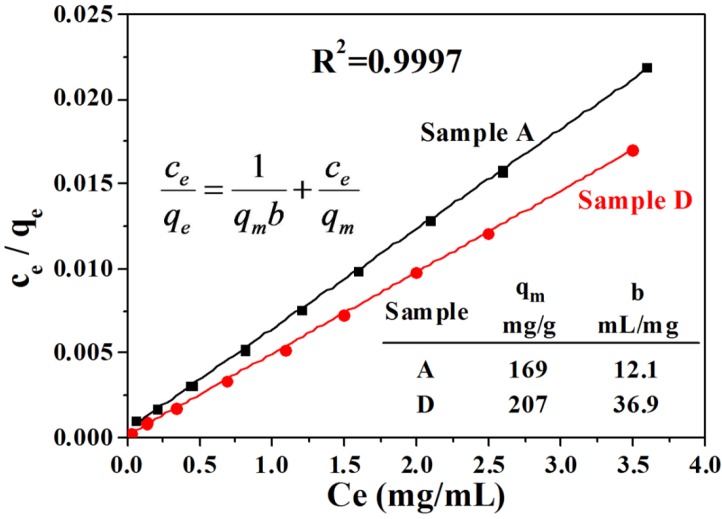
The Hb protein adsorption properties of Sample A (pH 5) and Sample D (pH 11). The linear relationship between *c_e_*/*q_e_* and *c_e_*.

As shown in Equation (1), where *q_e_* (mg·g^−1^) is the amount of Hb protein adsorbed at equilibrium, *c_e_* (mg·mL^−1^) is the equilibrium concentration of the solution, *q_m_* (mg·g^−1^) is the maximum adsorption capacity that corresponds to complete monolayer coverage and *b* is the equilibrium constant (mL·mg^−1^), the Langmuir isothermal adsorption equation represents the relationship between *q_e_* and *c_e_*. From Equation (2), one can see that the *c_e_*/*q_e_* has a linear relationship with *c_e_*. As shown in Figure 6, the experimental result also exhibited a good linear relationship between *c_e_*/*q_e_* and *c_e_*. According to the adsorption isotherms, the maximum Hb protein adsorption capacity *q_m_* of the Sample A and Sample D were 168.63 and 207.04 mg·g^−1^, and the corresponding equilibrium constant *b* were 12.1 and 36.9 mL·mg^−1^, respectively. The Hb protein adsorption capacity values of the Sample A (155 mg·g^−1^) and Sample D (205 mg·g^−1^) measured from experiment are close to the calculated values (168.63 and 207.04 mg·g^−1^) from the Langmuir adsorption model. Sample A was prepared with the microwave-assisted hydrothermal method at pH = 5 and consisted of HAP nanosheet-assembly flower-like hierarchical nanostructures. Meanwhile, Sample D, with a higher Hb protein adsorption capacity, was prepared with the same method at pH = 11 and consisted of mixed morphologies of nanorods and nanoprisms. The higher Hb protein adsorption capacity of Sample D may be explained by its smaller size and dispersed structure, compared with Sample A. The smaller size and dispersed structure may supply more adsorption sites for the protein molecules, and lead to the higher Hb protein adsorption capacity.

**Figure 7:**
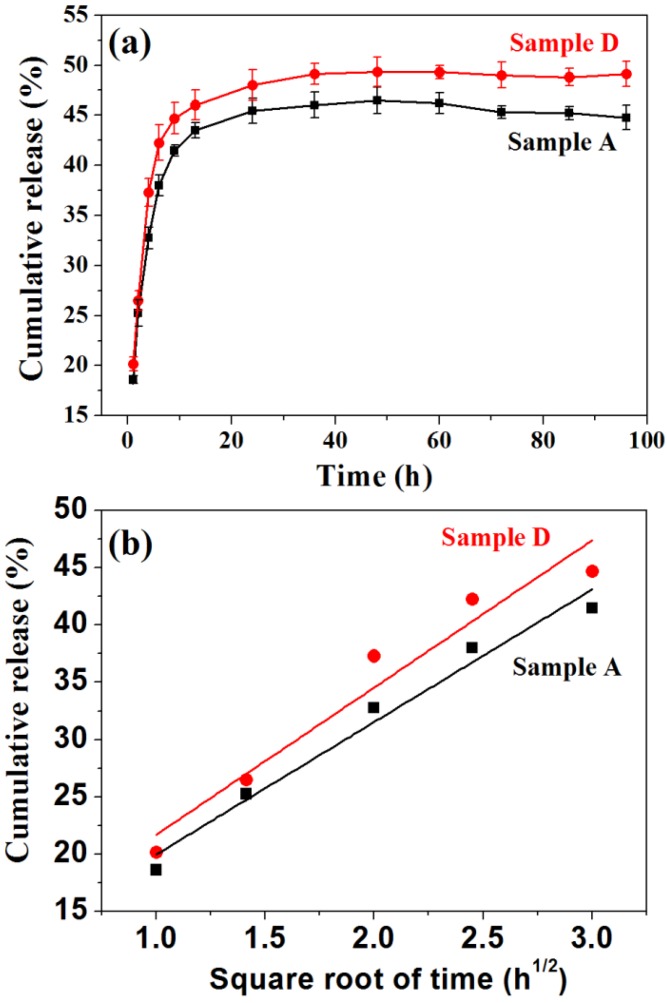
The Hb release properties of Sample A and Sample D. (**a**) Hb-release curve in PBS solution at different times; (**b**) cumulative Hb-release percentage as a function of the square root of the release time.

Figure 7a shows the Hb release behaviors of Hb-adsorbed Sample A and Sample D prepared in an Hb solution with a concentration of 2 mg·mL^−1^. The cumulative Hb release percentages from the Hb-adsorbed Sample A in PBS can reach 18.6%, 25.2%, 32.8% and 38.0% at release times of 1, 2, 4 and 6 h, respectively. Meanwhile, the cumulative Hb release percentages from the Hb-adsorbed Sample D can reach to 20.2%, 26.5%, 37.3% and 42.3% at release times of 1, 2, 4 and 6 h, respectively. The Hb release from both samples is rapid in the first several hours. After the initial rapid release stage, the Hb release rate can gradually decrease. The cumulative amounts of Hb released from Sample A and Sample D reach plateaus of 45% and 48% after 24 h, respectively. Moreover, for both Sample A and Sample D, the cumulative amount of released Hb has a good linear relationship with the square root of the release time, which is shown in Figure 7b. This result indicates that Hb release from Hb-adsorbed Sample A and Sample D is essentially governed by a diffusion process according to the Higuchi model [38,39].

## 3. Experimental Section

### 3.1. Preparation of Calcium Phosphate Nanostructures

In a typical experiment, 1.791 g of Na_2_HPO_4_·12H_2_O was dissolved in 250 mL of deionized water to form Solution A; 1.762 g of Ca(CH_3_COO)_2_·H_2_O and 0.26 g of 5-Fluorouracil was dissolved in 100 mL of deionized water to form Solution B. Thereafter, 10 mL of Solution B was added dropwise to 30 mL Solution A under magnetic stirring, and the pH values were adjusted to 5 (Sample A), 7 (Sample B), 9 (Sample C) and 11 (Sample D) by 1 M HCl or 1 M NaOH solution. Then, each resulting solution was put into an autoclave (60 mL), sealed and microwave-heated in a multimode-microwave oven (MDS-6, Sineo, Shanghai, China) to 120 °C and maintained at this temperature for 10 min under a heating power of 400 W. The multimode-microwave oven used in this study was a commercial instrument for the microwave-assisted liquid phase reaction. The temperature/pressure and the heating power were controlled by the instrument. After cooling down to room temperature, the products were separated by centrifugation, washed with deionized water and ethanol several times, and then dried to powder at 60 °C for 24 h. All of the chemicals used in the sample preparation were analytical grade, purchased from Sinopharm Chemical Reagent Co. (Shanghai, China), and used as received without further purification.

### 3.2. Characterization of Samples

The crystal phases of the as-prepared CaP samples were characterized using XRD recorded using a X-ray diffractometer (Rigaku D/max 2550 V, Cu Kα radiation, λ = 1.54178 Å) (Rigaku Co., Tokyo, Japan). FTIR spectra were taken on a FTIR spectrometer (FTIR-7600, Lambda Scientific, Edwardstown, Australia) using KBr pellets. The micrographs were taken with transmission electron microscope (TEM, Hitachi H-800, Tokyo, Japan).

### 3.3. Protein Adsorption and Release Properties

Hemoglobin (Hb) was chosen as a model protein for our investigation, and was purchased from Sangon Biotech (Shanghai, China). The protein-adsorption experiments at different protein concentrations were performed as follows: 5 mg of powdered CaP samples which were prepared with the microwave-assisted hydrothermal method under pH values of 5 (Sample A) and 11 (Sample D) were immersed in aqueous solutions that contained various concentrations of the protein (2 mL, 0.2–4.0 mg·mL^−1^). Each solution was treated ultrasonically for 10 min, and shaken at a constant rate (120 rpm) at 37 °C for 4 h. Then, the solution was centrifuged and the amount of protein in the supernatant was measured by UV/Vis absorption at a wavelength of 405 nm using a UV-Vis spectrophotometer (UV-2300, Techcomp, Shanghai, China).

For the *in vitro* protein release, 80 mg of powdered Sample A and sample D were immersed in an aqueous solution of Hb (2 mg·mL^−1^, 20 mL). Then, the solution was treated ultrasonically for 10 min, and continuously shaken in a sealed vessel at 37 °C for 4 h, followed by centrifugation and freeze drying to obtain the protein-adsorbed samples. The *in vitro* protein-release experiments were performed as follows: The powdered samples (8 mg) were immersed in phosphate buffered saline (PBS) solution (8 mL) (pH 7.4) at 37 °C with constant shaking (140 rpm). The protein-release solution (400 mL) was withdrawn for UV-Vis absorption analysis at given time intervals, and replaced with the same volume of fresh PBS (37 °C, pH 7.4).

## 4. Conclusions

Synthetic CaP materials have been considered ideal biomaterials due to their similar chemical structure to the native mineral components in the bones and teeth of vertebrates. Therefore, the preparation, control over structure/size/morphology, properties and applications of synthetic CaP nanostructures have become a hot topic. Herein, we have investigated the important role of pH value on the crystal phase and morphology of the CaP nanostructures prepared with the microwave-assisted hydrothermal method. The HAP nanosheet-assembled hierarchical nanostructures can be prepared under weak acidic conditions (pH 5), while the HAP nanorods can be prepared under neutral (pH 7) and weak alkali conditions (pH 9). When the pH value is increased to 11, the product consisted of a mixture of HAP nanorods and TCP nanoparticles. We can therefore regulate the morphology and structural properties of the CaP by adjusting the pH value. In addition, the as-prepared CaP nanostructures were investigated for potential applications in protein adsorption by Hb as a model protein. The sample which was prepared at pH = 11 and consisted of mixed morphologies of nanorods and nanoprisms showed a higher Hb protein adsorption capacity than the sample prepared at pH 5, which could be explained by the smaller size and dispersed structure. The experimental Hb protein adsorption capacity values of the samples prepared at pH 5 and pH 11 were 155 mg·g^−1^ and 205 mg·g^−1^, which are close to the calculated values (168.63 mg·g^−1^ and 207.04 mg·g^−1^) from the Langmuir adsorption model. The results reveal the as-prepared CaP nanostructures have a relatively high protein adsorption capacity, which is promising for applications in various biomedical fields such as drug delivery and protein adsorption.
